# Hemorrhagic Pericardial Effusion Secondary to Coxsackie B Pericarditis

**DOI:** 10.7759/cureus.76861

**Published:** 2025-01-03

**Authors:** Nolan Shoukri, Hazem Alakhras, Kateryna Strubchevska, Steven Timmis

**Affiliations:** 1 Internal Medicine, Oakland University William Beaumont School of Medicine, Rochester, USA; 2 Internal Medicine, Corewell Health William Beaumont University Hospital, Royal Oak, USA; 3 Cardiovascular Medicine, Corewell Health William Beaumont University Hospital, Royal Oak, USA

**Keywords:** coxsackie b, echocardiogram, hemorrhagic pericardial effusion, pericarditis, viral pericarditis

## Abstract

Acute pericarditis is caused by inflammation of the pericardial sac. Among the vast number of potential causes, viruses tend to trigger pericarditis most frequently. Some of the more common viral causes are Coxsackie A/B, echovirus, adenovirus, cytomegalovirus, herpes simplex virus, and human immunodeficiency virus. Pericardial effusion is a common complication and can be visualized on echocardiogram. In some cases, the pericardial effusion can be hemorrhagic in nature, which is extremely rare in the setting of viral pericarditis. The most common causes of hemorrhagic effusion are myocardial infarction, trauma, aortic dissection, or coronary artery bypass graft surgery. Pericardial effusion can sometimes result in serious complications such as cardiac tamponade. In cases of significant pericardial effusion, pericardiocentesis may be required. We present an interesting case of pericarditis caused by the Coxsackie B virus, causing significant hemorrhagic pericardial effusion requiring pericardiocentesis in a young patient. A 37-year-old female with no relevant past medical history presented with substernal chest pain radiating to the left arm and shoulder that improved with leaning forward and dyspnea for two weeks. She had a two-week history of a cough, dysphagia, fever, and chills that started two days prior to the presentation. EKG showed widespread ST elevations and PR interval depressions, which is consistent with a diagnosis of pericarditis. A large pericardial effusion was present on echocardiogram, further suggesting possible pericarditis. Around 350 mL of fluid was removed by pericardiocentesis. Cell count showed 201,000 red blood cells (RBCs)/mcL and 9,350 nucleated cells/mcL. Cytology was negative for malignancy. Cultures were negative for bacteria and fungi. Serum serology showed elevated inflammatory markers (C-reactive protein of 140 mg/L and erythrocyte sedimentation rate of 112 mm/hr) and increased Coxsackie B antibody titers (1:160 for type 2 and 1:320 for type 3). She was started on non-steroidal anti-inflammatory drugs and colchicine. This is a unique case showing that while small exudative pericardial effusions may occur with viral pericarditis, viral infections can also cause a significant hemorrhagic pericardial effusion. Most Coxsackie virus infections are benign. However, there are a few documented case reports of hemorrhagic pericardial effusion from Coxsackie B causing tamponade and death. The importance of this case is that it highlights the consideration of viral infections such as Coxsackie B as a potential cause of hemorrhagic tamponade, especially during autumn and winter months, seasons with the highest risk.

## Introduction

The pericardial sac is a fluid-filled cavity surrounding the heart, holding around 15-50 mL of ultrafiltrate plasma in a normal person. Due to pathology, the pericardial sac can be filled with excess fluid including transudate, exudate, or sanguineous fluid, which is described as a pericardial effusion. Causes of excess fluid accumulation can include infection, inflammation, malignancy, or direct filling of blood. If fluid buildup is large (>100 mL), cardiac tamponade can develop, which is an emergent condition where fluid build-up in the pericardial sac leads to compression of the heart and decreased cardiac output and shock. Development of cardiac tamponade can depend on the time period of fluid accumulation. Pericardial effusions that develop over long periods of time, such as in chronic conditions, accumulate a larger amount of fluid before causing significant impairment of cardiac function.

Inflammation of the pericardial sac is referred to as pericarditis. Pericarditis is categorized into idiopathic (where a cause is often not determined), infectious, and non-infectious. When a cause is determined, the most common cause of acute pericarditis (pericarditis lasting less than four to six weeks) is infection, which can occur from a wide variety of microbes. In developed countries, viral infection is the most common cause. However, in developing countries and worldwide, tuberculosis remains a significant cause of acute pericarditis. Studies conducted in the late 20th century have reported coxsackie A and B, adenovirus, and echovirus to be the more common causes of viral pericarditis. More recent studies have shown cytomegalovirus (CMV), herpes simplex virus (HSV), and human immunodeficiency virus (HIV) to be increasing in prevalence [1-2]. In some cases, the pericardial effusion can be hemorrhagic in nature, which is extremely rare in the setting of viral pericarditis. Common causes of hemorrhagic effusion include myocardial infarction, trauma, aortic dissection, or coronary artery bypass graft surgery [3].

On presentation, the patient may have chest pain that often improves with leaning forward and is more painful when lying supine, and physical examination can classically reveal a pericardial friction rub, a scratchy sound heard when auscultating the left sternal border. Often with viral etiologies, the patient can experience signs of a viral infection prior to the onset of chest pain. Overall, symptoms have a rapid onset and last up to four weeks. Initial testing includes electrocardiogram (EKG), chest X-ray, labs, and echocardiogram. EKG can initially show widespread ST elevations and PR depressions before normalizing after a few weeks, in which T wave inversion is seen that resolves after another few weeks to baseline [4]. However, this is not always present depending on the time since onset of disease. Electrical alternans can also present if a significant pericardial effusion forms. Chest X-ray is often normal but can show a large cardiac silhouette with large pericardial effusions. Echocardiogram can often help visualize if a pericardial effusion forms, as it is a common secondary complication to acute pericarditis. Labs can show signs of myocardial damage, such as increased troponin I or T, increased inflammatory markers such as C-reactive protein (CRP), erythrocyte sedimentation rate (ESR), or white blood cell (WBC) count.

Management of pericarditis includes both procedural and medical management. For diagnostic and therapeutic reasons, we can perform a pericardiocentesis, which is a procedure where a needle is used to remove excess fluid from the pericardial sac, and send the fluid for further studies. We can also leave a drain following the procedure to prevent build-up of pericardial fluid. Medical management includes non-steroidal anti-inflammatory drugs (NSAIDs) or aspirin, colchicine, and glucocorticoids. NSAIDs with colchicine are often the initial treatment, especially for viral pericarditis. Prednisone is a viable option for situations in which colchicine cannot be given or tolerated. In cases such as post-myocardial infarction pericarditis, aspirin is preferred to avoid inhibition of cardiac remodeling and preserve vessels. If the patient does not respond within a week, then further diagnostic evaluation should be conducted to find out the cause of the current illness. Colchicine is often given as an effective adjunct to NSAIDs to help with disease prognosis and reduce recurrence rate. If the patient has contraindications or cannot tolerate the initial regimen, then glucocorticoids can be introduced, but they are not preferred initially due to their increased risk of recurrence of pericarditis [1,5].

## Case presentation

A 37-year-old female with a past medical history of C-section, gastroesophageal reflux disease, and esophagitis presented to the emergency department with the chief complaints of chest pain and shortness of breath. The chest pain started two weeks prior but was the worst at presentation, with the patient rating it a 10/10. Pain was substernal, radiating to the left shoulder and arm, and was associated with shortness of breath, and symptoms were worse on lying down and better on sitting up. She reported a two-week history of dysphagia and a minor cough, with fever and chills that occurred two days prior. The patient denied any recent illnesses, headaches, abdominal pain, nausea, or vomiting, and has no known family history. Surgical history includes a colonoscopy and past C-section. Social history is notable for smoking (less than one pack per day), social drinking, and marijuana use daily. The patient was sexually active with one male partner. The patient was on fluticasone nasal spray prior to admission but no other medications.

Vitals on admission were notable for tachycardia with a heart rate of 105-116 beats/minute, respiratory rate of 20-23 breaths/minute, oxygen saturation of 93-98%, and blood pressure of 100-130 mmHg systolic. The patient was afebrile. Physical examination was notable for the patient being uncomfortable at rest, normal S1 and S2 heart sounds, tachycardia with regular rhythm, no jugular venous distention, no murmurs, lungs clear bilaterally to auscultation, normal respiratory effort, and a soft, non-distended abdomen with mild epigastric tenderness.

Labs

**Figure 1:**
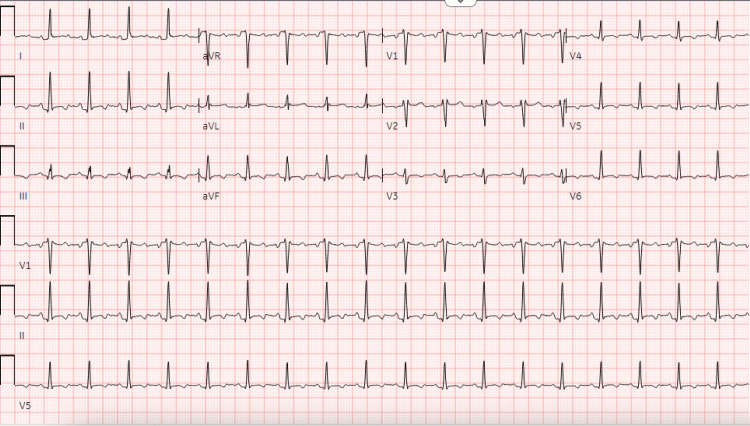
Prior EKG This is an EKG taken prior to the first appearance of electrical changes due to acute pericarditis.

**Figure 2:**
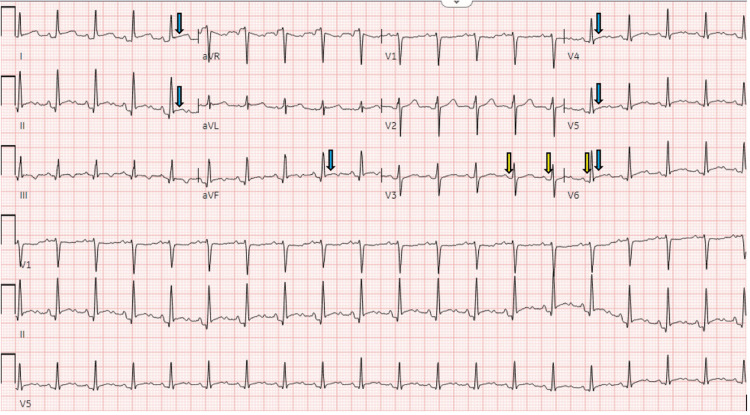
EKG showing acute pericarditis EKG showing normal sinus rhythm with widespread ST segment elevations (blue arrows) and PR interval depressions (yellow arrows), which was more apparent when comparing it to previous normal EKGs. Both changes were easier to identify when compared to previous EKGs.

**Figure 3:**
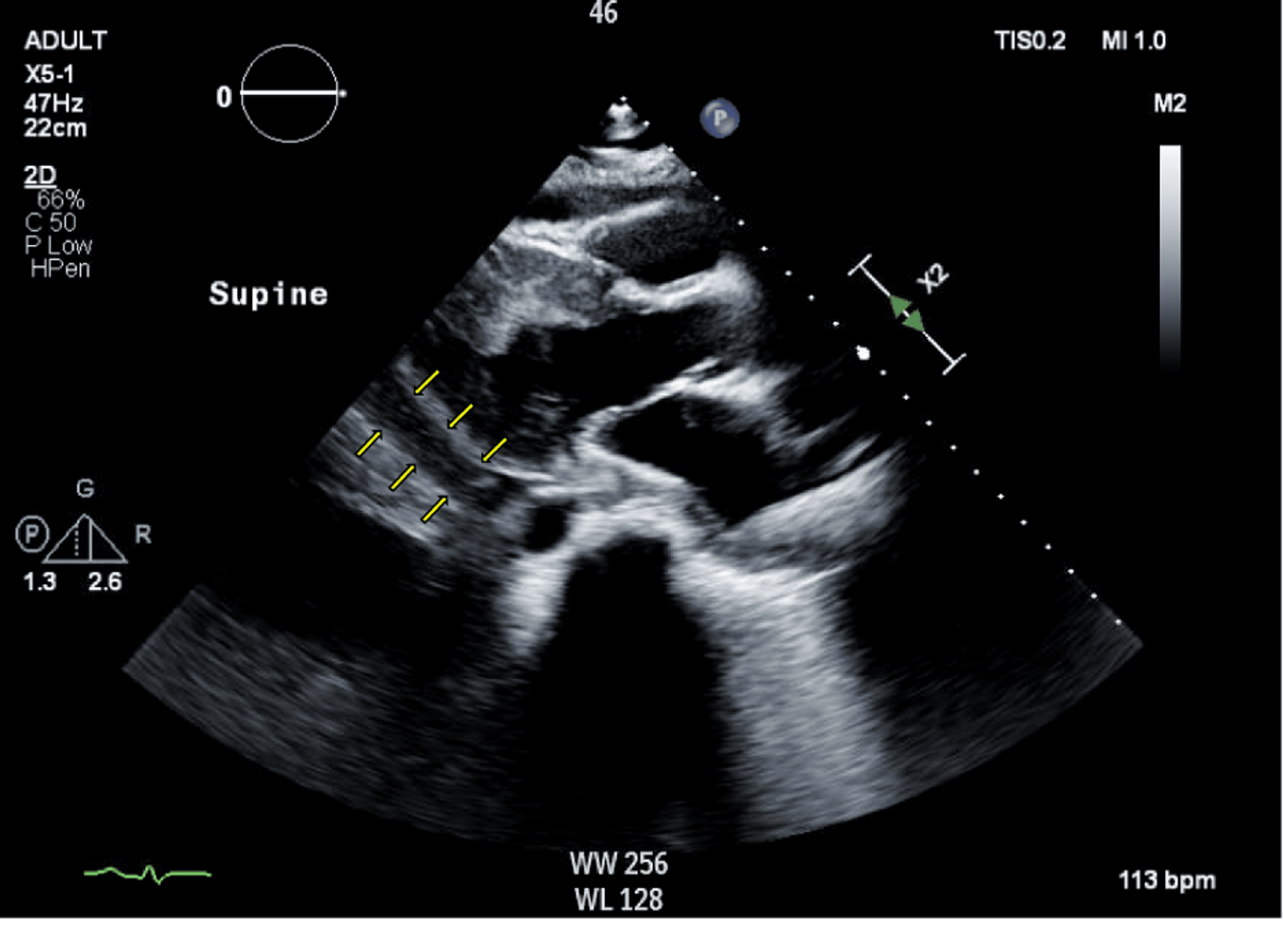
Echocardiogram showing pericardial effusion Echocardiogram showing pericardial effusion (yellow arrow). Fluid collection was followed throughout the hospital stay to determine resolution of the pericardial effusion.

**Figure 4:**
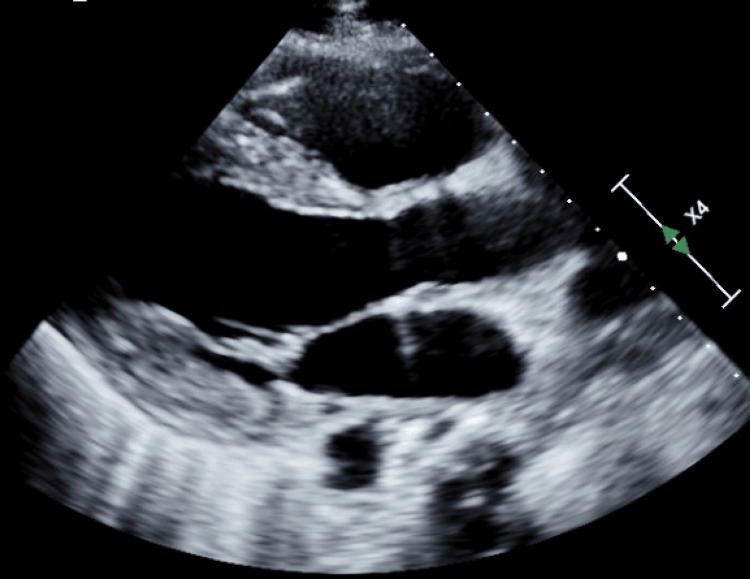
Post-procedure echocardiogram Echocardiogram taken a week following the pericardiocentesis, prior to discharge.

Initial labs were completed, with the most notable lab results are included in Table 1. All other initial testing was negative. Initial EKG was nondiagnostic with sinus tachycardia with nonspecific abnormalities. In comparison to a prior EKG (Figure 1), follow-up EKG the next morning (Figure 2) showed widespread ST segment elevations and PR interval depressions suggesting pericarditis. An echocardiogram was performed on day 2, demonstrating severely increased concentric wall thickness and large pericardial effusion (Figure 3) without hemodynamic compromise. As there was no hemodynamic instability, a diagnostic non-therapeutic pericardiocentesis was performed on day 2. Around 350 mL of fluid was removed. Cytology notable for a high number of RBCs. A cell count of the pericardial fluid was found to be cloudy, bloody fluid with 201,000 RBCs/mcL, and 9,350 nucleated cells/mcL with 81% neutrophils, 15% macrophages, and 4% monocytes out of 100 cells counted. Cultures of the pericardial fluid were negative for aerobic, anaerobic, fungal, and acid-fast bacilli. Follow-up echocardiograms on hospital days 5-7 showed no return of the pericardial effusion. Echocardiograms performed on hospital days 8 and 9 (Figure 4), which showed mild pericardial effusion and left pleural effusion. Follow-up infectious and serology studies that were notable are included in Table 1, and all other labs were not notable.

**Table 1:** Initial and follow-up labs Many labs were conducted throughout the patient's entire hospital stay, including ruling out many other infectious or autoimmune causes of her presentation. Only the most notable labs were included in this table. CRP, C-reactive protein; ESR, erythrocyte sedimentation rate

Notable Labs	Patient Value	Reference Range
Initial labs		
White blood cell count	15.9 bil/L	3.3-10.7 bil/L
Hemoglobin	11.3 g/dL	12.1-15 g/dL
Hematocrit	34.7%	35.4-44.2%
Sodium	134 mmol/L	135-145 mmol/L
Bicarbonate	18 mmol/L	20-29 mmol/L
ESR	64 mm/Hr	0-18 mm/hr
CRP	229 mg/L	<8.0 mg/L
High-sensitivity troponin I	<4 ng/L	<17 ng/L
Respiratory viral panel	Not detected	Not detected
Follow-up labs		
CRP	140.7 mg/L	<8.0 mg/L
ESR	112 mm/hr	0-18 mm/hr
Coxsackie B antibodies	1:160 type 2 ; 1:320 type 3	<1:10
Anti-nuclear antibody	1:160	<1:160
Fungitell®	45 pg/mL	<59 pg/mL
Ebstein-Barr virus viral capsid antigen antibodies	IgG 247 U/mL, IgM 18.4 U/mL	IgG 0-17.9 U/mL, IgM 0-35.9 U/mL

Management

**Figure 5:**
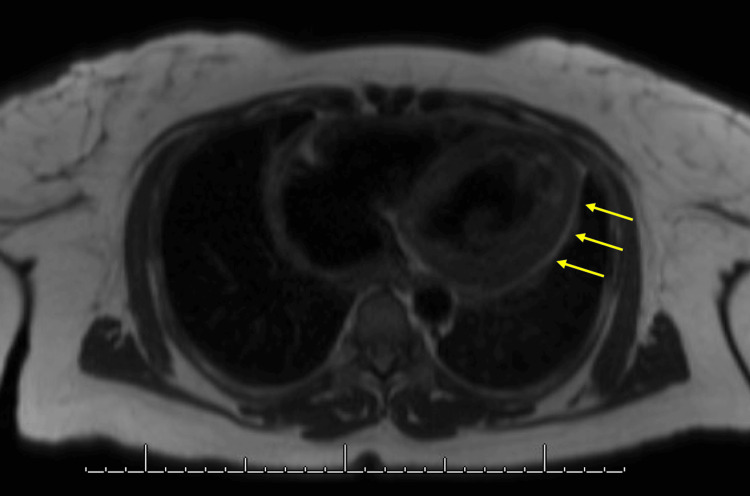
Post-hospitalization cardiac MRI Cardiac MRI taken following discharge from the hospital. The yellow arrows highlight the thickening of the pericardium.

The patient’s presentation, labs, and procedure results made pericarditis most likely as a differential diagnosis, which resulted in plans to investigate whether it was caused by autoimmune, infectious, or postcardiac injury. Other diagnoses considered on the differential were malignancy, post-myocardial infarction, drugs, or ovarian hyperstimulation syndrome. Since the patient’s EKG findings showed pericarditis, and her echocardiogram showed pericardial effusion, a pericardiocentesis was performed for fluid evacuation. A drain was left in until hospital day 8, and she continued her management in the critical care unit. After the pericardiocentesis, repeat echocardiograms showed minimal return of fluid with a mild effusion prior to discharge. The pericarditis was initially treated with ibuprofen 800 mg thrice per day and colchicine 0.6 mg twice a day. Prednisone 20 mg daily was added and colchicine was reduced when she was not able to tolerate the colchicine due to diarrhea. She was discharged on hospital day 11. Cardiac magnetic resonance imaging was performed after discharge, which showed 6mm pericardial thickness, trivial pericardial effusion, and pericardial inflammation without myocardial involvement (Figure 5). The lack of myocardial involvement provides insight into the negative high-sensitivity troponin labs. The patient was recommended to follow-up outpatient to ensure full resolution of symptoms and for further investigation of any autoimmune causes of her presentation.

## Discussion

On presentation, the patient’s signs and symptoms initially worried the team about cardiac involvement, leading to her extensive labs and EKG. Once the EKG showed pericarditis as the possible cause of the patient’s presentation, further lab work ruled out many of the causes of the current presentation. The patient’s history did not include any pertinent information to help explain what risk factors could have led to this patient’s presentation, as there was no history of autoimmune disease, myocardial infarction, chronic kidney disease, or infections prior to the onset of the chest pain. Labs ruled out urgent causes such as a myocardial infarction. Patient history also ruled out some possible etiologies of pericarditis such as uremia, trauma, and drug-induced inflammation.

Echocardiogram showed a pericardial effusion. Diagnostic pericardiocentesis removed a significant amount of fluid, which was sent for cultures and polymerase chain reaction (PCR) to identify the cause of the pericarditis. Infectious and autoimmune etiologies could not be ruled out, which necessitated further investigation outpatient. Treatment with ibuprofen and colchicine was started for the patient. Prednisone was initiated due to intolerance of full-dose colchicine. Prednisone is not the preferred first line for treatment. However, for patients not able to tolerate colchicine, glucocorticoids are a suitable addition. The patient was followed for a few days for recurrence of symptoms and the pericardial effusion before being discharged with outpatient follow-up. Based on current literature regarding management of pericarditis, it appears that the patient was managed appropriately, and the appropriate follow-up was set in place to continue care for the patient [1-2,5].

Pericardial effusion is something that commonly occurs with viral pericarditis. This case is an interesting example of how Coxsackie B virus, which generally has a benign course, can lead to large hemorrhagic pericardial effusions. Coxsackie B pericarditis is more prevalent in the autumn and winter. If left untreated, this hemorrhagic effusion could develop into a cardiac tamponade. Cardiac tamponade due to viral pericarditis is very rare. However, it has been reported in the past [6,7].

A limitation of this case is that a PCR was not conducted. However, serology was consistent with a current Coxsackie B infection, which is a common cause of pericarditis and was determined to be the most likely source of the pericardial effusion. Autoimmune causes are other possible sources to investigate as we saw with an increased antinuclear antibody.

## Conclusions

Based on our patient’s presentation and results from labs and procedures, Coxsackie B pericarditis leading to hemorrhagic pericardial effusion ended up being the final diagnosis. While pericarditis is often idiopathic, the majority of cases in developed countries tend to be caused by viral infection. Coxsackie B is a common virus leading to pericarditis. It is important to keep disease progression, such as this case, in mind because while it might be rare, it can lead to extremely poor patient outcomes if not managed properly.
